# Scoring system for prediction of overall survival in patients with renal cell carcinoma T3aN0M0

**DOI:** 10.1002/bco2.309

**Published:** 2023-11-10

**Authors:** Caio Vinícius Suartz, Maurício Dener Cordeiro, Paulo Afonso de Carvalho, Fábio Pescarmona Gallucci, Leopoldo Alves Ribeiro‐Filho, Leonardo Cardili, Arjun Sivaraman, François Audenet, José Mauricio Mota, William Carlos Nahas

**Affiliations:** ^1^ Department of Urology, Hôpital Européen Georges Pompidou Université Paris Cité Paris France; ^2^ Division of Urology, Instituto do Cancer do Estado de São Paulo University of São Paulo São Paulo Brazil; ^3^ Department of Urology Washington University in St. Louis St. Louis Missouri USA; ^4^ Genitourinary Medical Oncology Service, Instituto do Cancer do Estado de São Paulo University of São Paulo São Paulo Brazil

**Keywords:** adjuvant therapy, kidney neoplasms, oncologic outcome, prognostic factor, renal cell carcinoma

## Abstract

**Objective:**

We aim to create a new score to predict postoperative overall survival in patients with nonmetastatic T3aN0 renal cell carcinoma.

**Methods:**

We reviewed the clinical data of adult patients who underwent radical nephrectomy for renal cell carcinoma between December 2007 and January 2022 in a single tertiary oncological institution. Clinical characteristics, clinical‐pathological staging and histopathological characteristics were analysed. Survival analyses were determined using the Kaplan–Meier curve. A nomogram was established using Cox proportional hazard regression to identify the prognostic factors affecting the overall survival. The area under the curve, calibration curves and decision curve analysis were used to evaluate prognostic efficacy.

**Results:**

We analyzed 362 patients classified as pT3aN0M0 stage with a median follow‐up of 40 months. According to Cox univariate and multivariate analyses, weight loss greater than 5% in 6 months before surgery, stage V chronic kidney disease after radical nephrectomy, sarcomatoid pattern, and coagulative tumor necrosis were identified as predictors of overall survival. We developed a score and performed internal and external validation. The time‐dependent receiver operating characteristic curve, area under the curve value and calibration curve analysis showed good prediction ability of the score. The nomogram can effectively predict and stratify overall survival after radical nephrectomy in patients with pT3aN0M0 renal cell carcinoma.

**Conclusion:**

Patients with pT3aN0MO renal cell carcinoma exhibited different characteristics, and those with unfavourable characteristics deserve greater attention during follow‐up. This nomogram provides an accurate prediction of overall survival after radical nephrectomy.

## 1 INTRODUCTION

The incidence of renal cell carcinoma (RCC) has increased in the last 30 years. In the United States, there are approximately 63 000 new cases and 14 000 deaths each year.^1^ Imaging methods, such as computed tomography of the abdomen, have increased the early detection of these tumours. Correspondingly, paradigms for treating kidney cancer are changing with the advent of more effective systemic targeted therapies.^2^

The tumoural staging was created to distinguish neoplasms with the same pathological type but with different oncological outcomes in the postoperative follow‐up. The TNM staging system for RCC, developed by the American Joint Committee on Cancer, is one of RCC's best indicators of independent prognosis.^3^

Patients with renal tumours at the pT3aN0MO stage are grouped into a single prognostic category. However, clinical practice reveals that there may still be an under‐staging within this heterogeneous group of patients due to different oncological evolutions.^4^

This study aimed to analyse prognostic factors and overall survival (OS) in patients with T3aN0MO renal cell carcinoma who underwent radical nephrectomy and establish a prognostic nomogram. This may be valuable for improved treatment decisions to improve clinical outcomes.

## 2 METHODS

**FIGURE 1:**
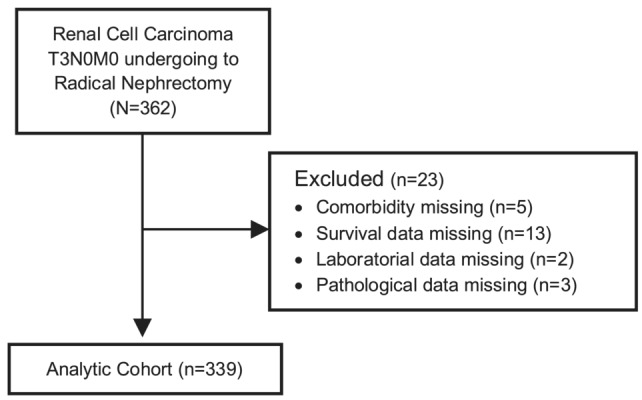
CONSORT flow diagram showing patient exclusions.

This study is a single‐centre retrospective cohort approved by study Institutional Review Board (1025/2016). We evaluated 739 patients who underwent radical nephrectomy for RCC between December 2007 and October 2022 at a tertiary cancer care centre. The patient's medical records were reviewed and identified. The American Joint Committee on Cancer 2017 classification^3^ was used to classify patients according to the pT3aN0M0 stage. All patients with other stages, positive lymph nodes or metastases were excluded (Figure 1).

### 2.1 Variables

We analysed the role of the following clinical and pathological factors: age, sex, body mass index (BMI), symptoms, co‐morbidities, weight loss (>5% in 6 months) before surgery, neutrophil‐to‐lymphocyte ratio (NLR), Charlson co‐morbidity index (CCI), lymphovascular invasion, renal sinus, and perirenal fat involvement, sarcomatoid pattern, presence of coagulative tumour necrosis and calculated eGFR with the Chronic Kidney Disease Epidemiology Collaboration (CKD‐EPI) formula using serum creatinine levels 6 months after surgery and classified with Kidney Disease Improving Global Outcomes (KDIGO).

### 2.2 Oncological outcomes

The primary study outcome was OS, which was calculated from the date of radical nephrectomy to the date of death or the last follow‐up. Patients with a survival time of 0 months were excluded. Survival status was determined by querying patient hospitalization data and telephone follow‐up. The end time of follow‐up was October 2022.

Recurrence‐free survival (RFS) and cancer‐specific survival (CSS) were estimated by the Kaplan–Meier method.^5^ Recurrence was defined as new radiological or pathological evidence of tumour relapse. CSS was considered the length of time from either the date of diagnosis or the start of treatment for a disease, such as cancer, to the date of death from the disease. Patients who die from causes unrelated to the disease are not counted in this measurement.

### 2.3 Statistical analysis

In the descriptive analysis, parametric variables are represented by mean and standard deviation, non‐parametric variables are represented by a median and interquartile interval, and proportion frequency as percentages. The confidence interval adopted was 95%, with a *p*‐value < 0.05 considered significant. The normality of the variables was tested using Kolgomorov–Smirnov and Shapiro–Wilk tests.

SPSS Statistics 28.0.1.1 was used to perform the statistical analyses. The univariate analysis was used to identify significant oncological outcome predictors. Cox proportional hazard regression analysis was used to determine the significant multivariate predictors of oncological outcomes. In a backward stepwise fashion, the significant univariate variable with minor significance was eliminated from the multivariable model until only the significant variables remained. Survival analyses were performed with Kaplan–Meier estimated curves, and pairwise comparisons using the log‐rank tests were performed.

A nomogram to predict postoperative overall survival was developed based on the independent risk factors identified in the multivariate analysis. The cohort was randomly divided 1:1 into development and validation sets. The predictive performance of the nomogram was analysed with Harrel's C‐index, and a calibration curve was used to verify the prediction effect.

Each independent prognostic factor in the nomogram was assigned a score. The log‐hazard ratio (HR) parameters (β) estimates from the initial model were used to estimate the relative contribution of each variable in predicting T3aN0M0 RCC overall survival. Variables with small maximum log‐HRs were dropped from the model, and a log‐HR of approximately 0.5 was associated with 1 point in the model. Next, the time‐dependent receiver operating characteristic (ROC) curve and the area under the curve (AUC) value were elaborated to estimate the prediction accuracy of the nomogram. The calibration curve and decision curve analysis were used to verify the prediction effect of the model. Then, we performed validation training for external validation to evaluate the performance of the prediction model.

## 3 RESULTS

A total of 362 patients with pT3a were included in the study. The mean age was 64.97 years, and 65% (*n* = 235) were male. After excluding patients with missing demographic, clinical, pathological or follow‐up data, the dropout rate was 6%, and 339 patients were analysed. After a median follow‐up of 40.2 months, recurrence occurred in 22.7% (*n* = 77), death in 26.0% (*n* = 71) and cancer‐specific death in 12.0% (*n* = 41) of patients (Tables 1 and 2).

**TABLE 1:** Clinical characteristics.

Clinical characteristics of 339 patients with pT3aN0MO RCC	
Characteristic	Overall population
Age (years)	
Mean (SD)	62.3 (12.1)
Gender, *N* (%)	
Male	212 (62.5)
Female	127 (37.4)
BMI (kg/m^2^), median (IQR)	27.5 (24.5–31.5)
Symptoms	
No symptoms, *N* (%)	148 (43.6)
Haematuria, *N* (%)	123 (36.2)
Abdominal pain, *N* (%)	104 (30.6)
Weight loss, *N* (%) (≥5% in the last 6 months or 10% in 1 year)	42 (12.3)
Co‐morbidities, *N* (%)	
Hypertension	203 (59.8)
Diabetes mellitus	92 (27.)
Charlson co‐morbidity index, *N* (%)	
Mild 1–2	94 (27.7)
Moderate 3–4	134 (39.5)
Severe ≥5	111 (32.7)
Stage V chronic kidney disease 6 months after radical nephrectomy, *N* (%)	11 (3.2)

**TABLE 2:** Pathological characteristics.

Pathological features	Overall population
Primary tumour size (cm)	8.5 (6.2–10.9)
Median (IQR)	
Fuhrman nuclear grade (%)	
1	0.3
2	28.7
3	51.6
4	19.4
Lymphovascular invasion (*n*, %):	
Yes	88 (25.9)
No	251 (74.0)
Coagulative tumour necrosis (*n*, %):	
Yes	171 (50.4)
No	168 (49.5)
Sarcomatoid differentiation (*n*, %):	
Yes	24 (7.6)
No	315 (92.3)
Renal sinus fat invasion (*n*, %):	
Yes	255 (75.2)
No	84 (24.7)
Perinephric fat invasion (*n*, %):	
Yes	126 (37.1)
No	212 (62.5)
Renal capsule invasion (*n*, %):	
Yes	110 (32.4)
No	229 (67.5)
Thrombus in renal vein (*n*, %):	
Yes	55 (16.2)
No	284 (83.7)

### 3.1 Univariate predictors

The significant predictors of RFS were tumour size ([HR]: 1.02; 95% CI: 8.3–9.6, *p* < 0.001), Fuhrman grade ([HR]: 2.1; 95% CI: 2.7–2.9, *p* < 0.001), weight loss ([HR]: 4.2; 95% CI: 2.1–8.3, *p* < 0.001), presence of coagulative tumour necrosis ([HR]: 2.8; 95% CI: 1.6–4.9, *p* < 0.001), sarcomatoid pattern ([HR]: 4.6; 95% CI: 1.9–10.8, *p* < 0.001), tumour invasion of the renal sinus fat ([HR]: 3.0; 95% CI: 1.4–6.3, *p* = 0.002), body mass index ([HR]: 1.1; 95% CI: 27.6–28.9, *p* = 0.036) and neutrophil lymphocyte ratio (NLR) ([HR]: 1.04; 95% CI: 2.7–3.3, *p* = 0.018).

The statistical significant predictors of CSS were tumour size ([HR]: 1.0; 95% CI: 0.9–1.0, *p* < 0.001), NLR ([HR]: 1.0; 95% CI: 0.9–1.1, *p* < 0.028), BMI ([HR]: 0.9; 95% CI: 0.8–0.9, *p* < 0.001), renal sinus fat invasion ([HR]: 1.5; 95% CI: 0.6–3.5, *p* < 0.040), sarcomatoid pattern ([HR]: 4.6; 95% CI: 2.2–9.5, *p* < 0.001), coagulative tumour necrosis ([HR]: 7.8; 95% CI: 3.0–20.4, *p* < 0.001), weight loss ([HR]: 3.5; 95% CI: 1.7–7.1, *p* = 0.002) and Fuhrman grade ([HR]: 2.1; 95% CI: 1.3–3.2, *p* < 0.001).

The statistical significant predictors of OS were tumour capsule invasion ([HR]: 1.02; 95% CI: 0.5–1.7, *p* = 0.02), tumour invasion of renal sinus fat ([HR]: 1.1; 95% CI: 0.5–1.8, *p* = 0.002), stage V chronic kidney disease ([HR]: 3.9; 95% CI: 1.4–10.9, *p* = 0.002), sarcomatoid pattern ([HR]: 5.5; 95% CI: 2.3–13.1, *p* < 0.001), coagulative tumour necrosis ([HR]: 3.2; 95% CI: 1.7–6.0, *p* < 0.001), weight loss ([HR]: 2.4; 95% CI: 1.1–5.0, *p* = 0.017) and Fuhrman grade ([HR]: 1.96; 95% CI: 1.2–2.9, *p* = 0.02).

### 3.2 Multivariate predictors of overall survival

**FIGURE 2:**
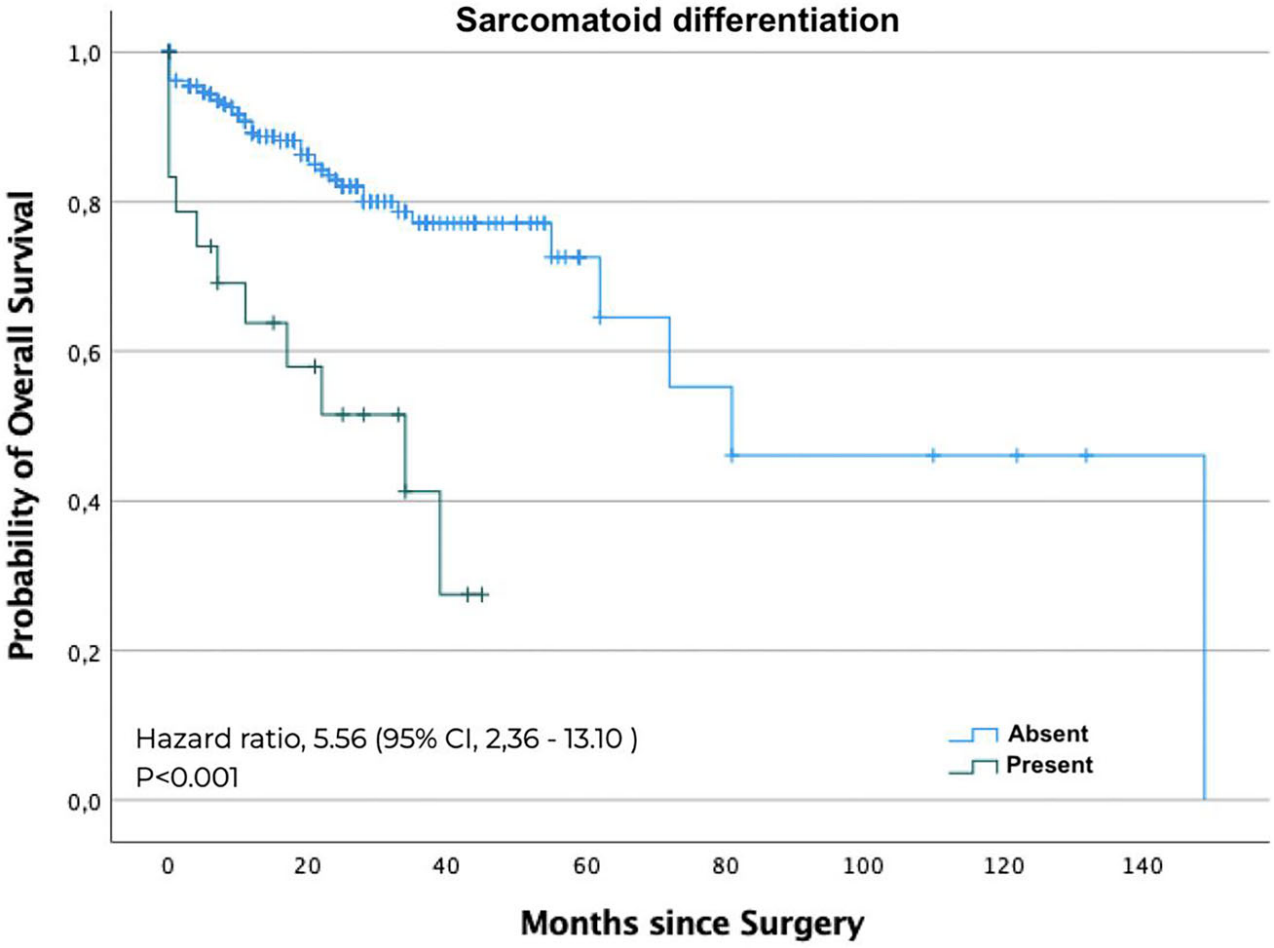
The Kaplan–Meier curve analysis for overall survival by sarcomatoid differentiation.

**FIGURE 3:**
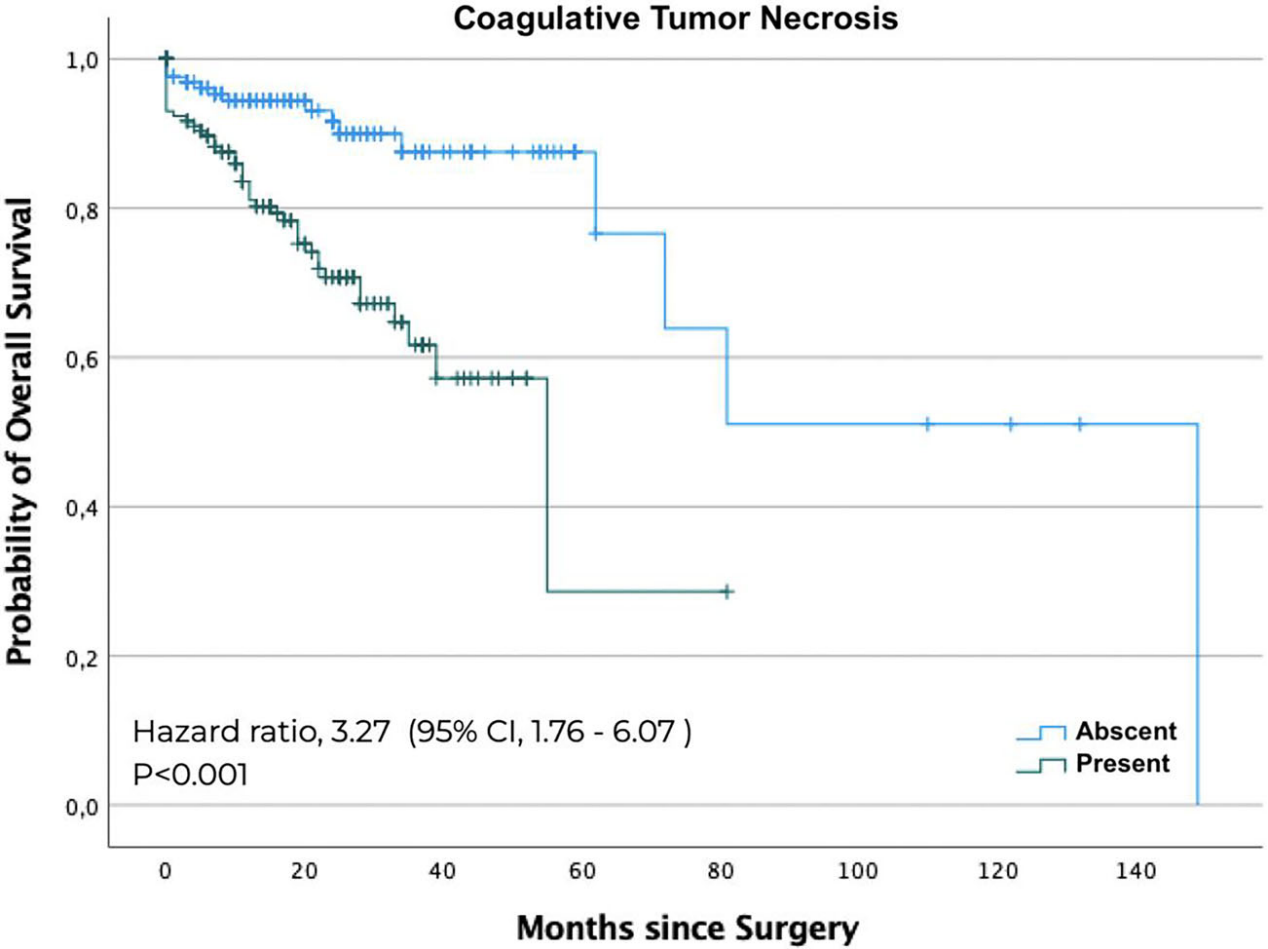
The Kaplan–Meier curve analysis for overall survival by coagulative tumour necrosis.

The Cox proportional hazards analysis showed that sarcomatoid pattern ([HR]: 5.56; 95% CI: 2.3–13.1, *p* < 0.001), coagulative tumour necrosis ([HR]: 3.27; 95% CI: 1.7–6.0, *p* < 0.001), weight loss greater than 5% in 6 months before surgery ([HR]: 1.6; 95% CI: 1.3–4.2, *p* = 0.005), stage V chronic kidney disease after radical nephrectomy ([HR]: 2.2; 95% CI: 1.2–5.0, *p* = 0.017) were associated with OS. The OS curves illustrate the difference between patients with and without the presence of a sarcomatoid pattern (Figure 2) and coagulative tumour necrosis (Figure 3). The predictors of global survival were studied in univariate analysis and multivariate analysis (Table S1).

### 3.3 Nomogram and score to predict overall survival in RCC T3aN0M0

**FIGURE 4:**
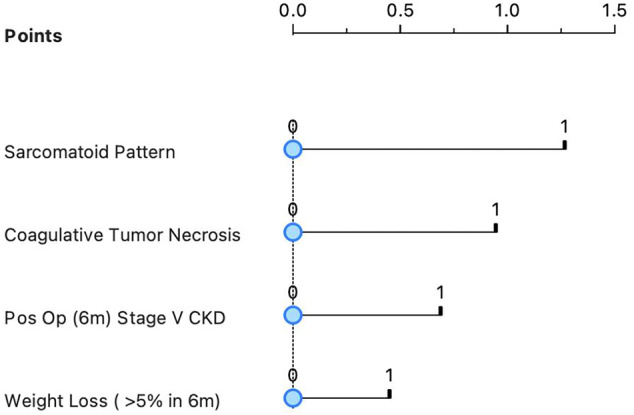
Nomogram to predict overall survival in T3aN0 renal cell carcinoma.

**FIGURE 5:**
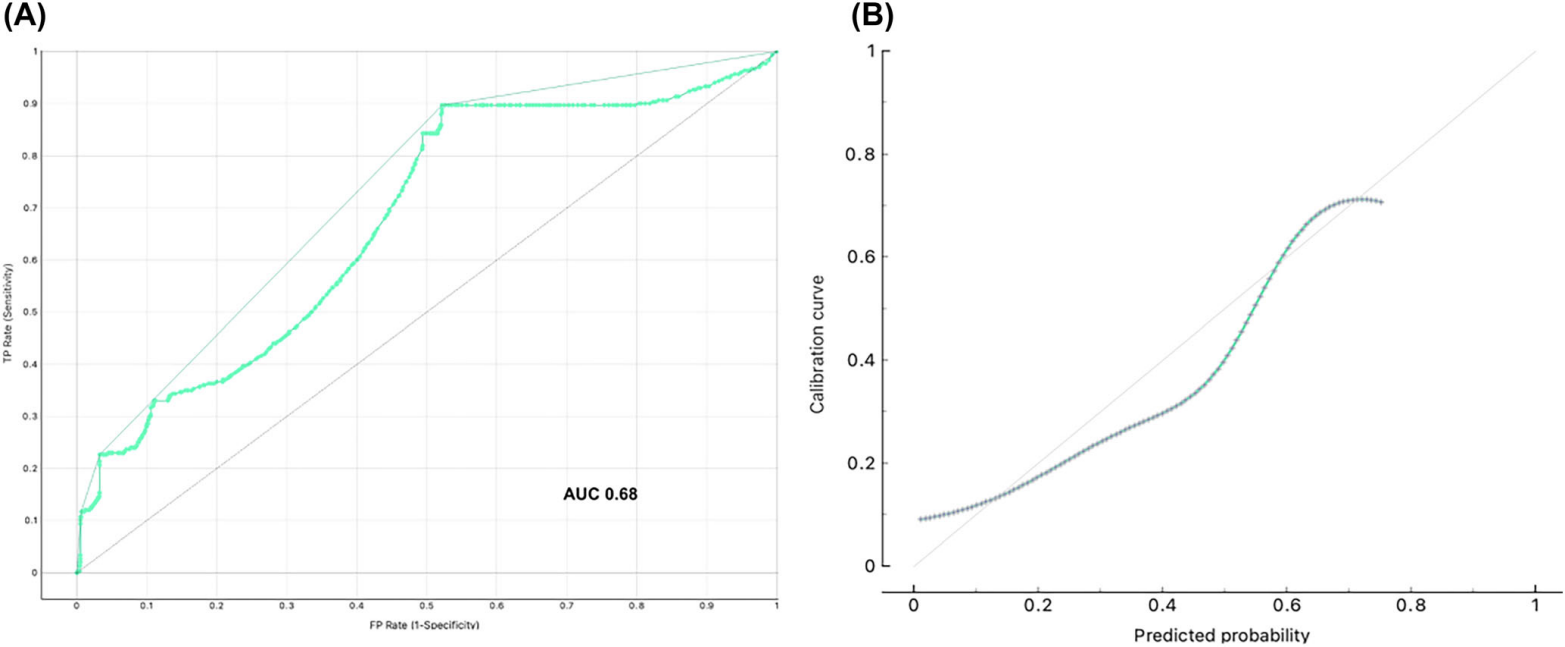
The receiver operating characteristic curve and area under the curve value (A) and calibration plot (B) of the nomogram for predicting 5‐year overall survival (OS) in the training cohort.

The 5‐year predictive nomogram of OS for patients with nonmetastatic T3aN0M0 RCC was established after incorporating the four significant predictors, which were identified in the multivariate analysis. The sarcomatoid pattern showed the maximum weight of the points, followed by coagulative tumour necrosis, stage V chronic kidney disease and weight loss (Figure 4). Using bootstraps with 1000 resamples, the C‐index of the nomogram was 0.68 (95% CI: 0.56–0.71) and the calibration curve of 5‐year OS presented consistency between the predicted and observed survival (Figure 5A,B).

**FIGURE 6:**
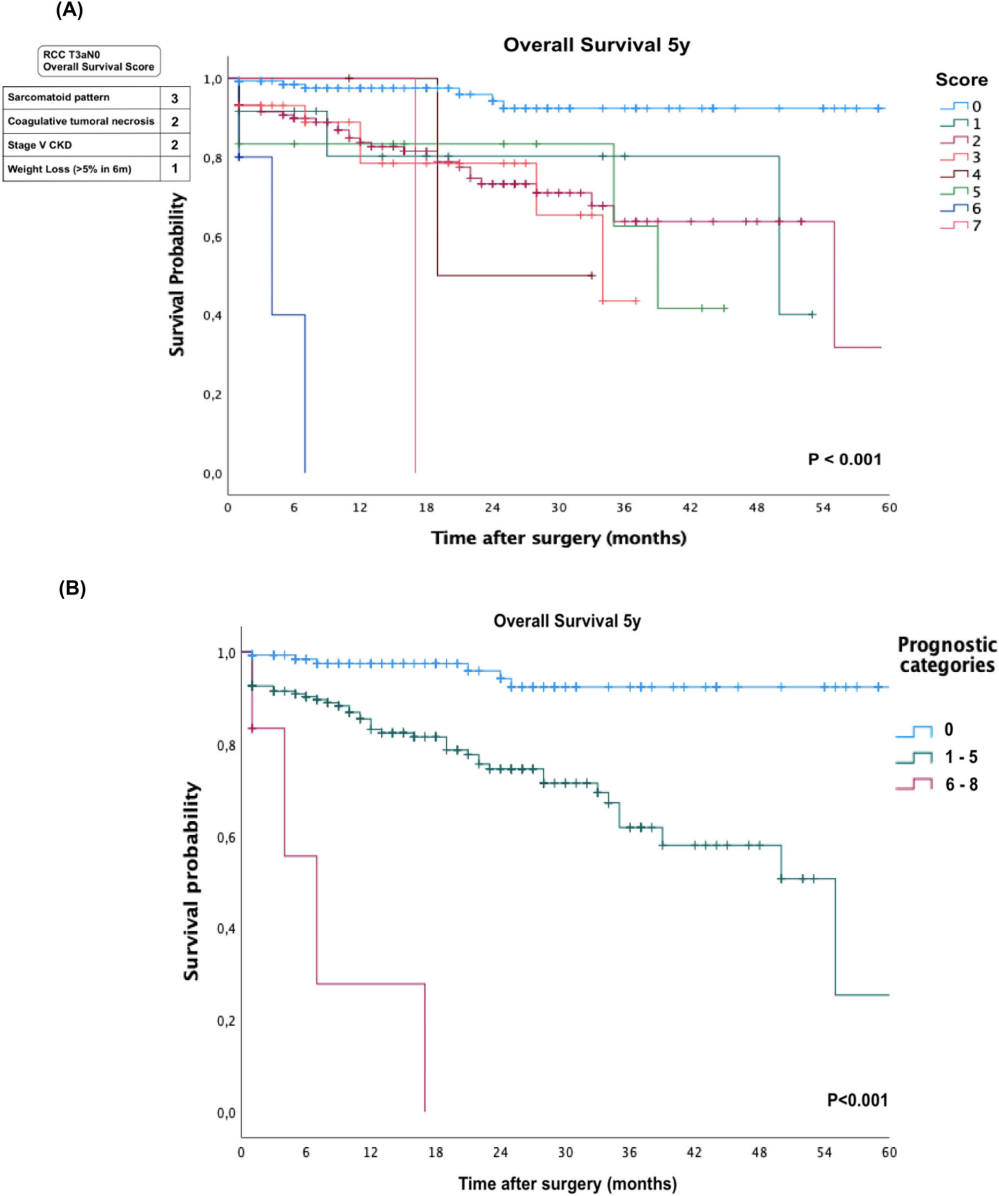
Score to predict overall survival in 5 years for patients with renal cell carcinoma (RCC) T3aN0 and Kaplan–Meier curve analysis with log‐rank test (A). Stratified score into three categories with logrank test (B).

The variables received scores to respect the proportionality of the beta coefficients and the predictive accuracy of the newly designed score was evaluated in the Kaplan–Meier survival analysis stratified by each score, and the significance of the log‐rank test was used to visually and statistically confirm that the score adequately stratified patients by observed survival (Figure 6A) The visual evaluation of the Kaplan–Meier curves allowed divided patients into three different prognostic groups (Figure 6B). Patients with score 0 points had a good prognosis, patients with scores between 1 and 5 points intermediate prognoses and patients with scores equal to or greater than 6 points a worse prognosis. This model presented a ROC curve with AUC of 0.7 demonstrating great discrimination of the score (Figure S1).

## 4 DISCUSSION

The current results demonstrate in a large cohort of patients with T3aN0M0 RCC who underwent radical nephrectomy, there was a wide range of expected survival. The newly created nomogram and prognostic score accurately stratify the risk of overall mortality in a large cohort.

The critical period for the development of tumour recurrence was 1.5 years after surgery. A total of 22.7% (77) of the patients presented recurrence; of these, 8.2% (28) died from kidney tumour‐related causes within 17.1 months. These data reinforce the importance of maintaining close follow‐up in the first 2 years after nephrectomy. Single and localized tumoural recurrences underwent surgical resections. Patients with disseminated recurrences with metastatic disease were managed with the standard systemic treatment available at the institution, Pazopanib, which is a multi‐target tyrosine kinase inhibitor of vascular endothelial growth factor receptors 1, 2 and 3 (VEGFR‐1, VEGFR‐2, VEGFR‐3), platelet‐derived growth factors α and β (PDGFR‐α and PDGFR‐β) and stem cell factor receptor (c‐KIT). We do not have immunotherapy or targeted therapy currently in the public health system.

Weight loss of 5% until 6 months before surgery was associated with worse overall survival and specific cancer survival. In the general population studied, 12.3% of the patients showed weight loss and a median BMI of 27.5 kg/m^2^ (range 24.5–31.5), whereas patients who died of kidney cancer presented a median BMI of 25 kg/m^2^ (range 22.7–28.0). Kim et al. studied patients with kidney cancer but with a less advanced stage (pT1N0M0) and reported a similar finding, with cachexia associated with RFS (HR: 3.0; *p* = 0.032) and CSS (HR: 4.3; *p* = 0.011).^6^ In another study, Morgan et al. analysed a retrospective cohort with 369 patients from 2003 until 2008 and presented weight loss (>5% in 6 months before surgery) as an independent prognostic factor associated with overall and cancer‐specific survival in patients who underwent radical or partial nephrectomy for RCC. The hazard ratio to overall survival was 2.41, similar to our study.^7^

The presence of isolated coagulative tumour necrosis was a predictor of CSS (*p* < 0.001) and OS (*p* = 0.003) in both the univariate and multivariate analyses. Coagulative tumour necrosis occurs when the growth of the tumour exceeds its blood supply, denoting aggressive tumour behaviour with rapid proliferation and progression,^8^ which explains the study's findings. Coagulative tumour necrosis in renal tumours as a marker of poor prognosis has also been reported in other studies.^9^, ^10^

Sarcomatoid patterns can be found in 5%–8% of RCC cases. This histological pattern confers aggressiveness, poor prognosis, and a high risk of recurrence.^8^, ^11^ Our study showed similar results to the literature,^8^, ^12^, ^13^ with the sarcomatoid pattern at higher risk of cancer‐specific death (HR: 5.6) and overall mortality (HR: 5.5) than the rest of the study population.

Patients with chronic kidney disease (CKD) stage V have higher overall mortality compared to the general population.^14^ In our study, we had 9 (2.6%) patients in the preoperative period with stage V CKD and 11 (3.2%) patients after 6 months from radical nephrectomy, and the mortality risk is inversely proportional to eGFR.^15^ Patients who underwent radical nephrectomy and who had previous chronic kidney disease had higher mortality than the general population, and the prognosis is equally poor for patients who developed chronic kidney disease after radical nephrectomy (RR: 1.4; 95% CI: 1.1–1.8).^16^

To our knowledge, it is the first score to stratify T3aN0M0 patients according to overall survival. The only published nomogram involving RCC T3a patients used the SEER database, included patients with positive nodes, presented a follow‐up time of only 8 years, and the primary outcome studied was specific cancer survival. The author recognizes that preoperative and postoperative renal function and the presence of tumour necrosis that was not recorded in the SEER database may have influenced the analysis and caused confounding factors.^17^

Finally, we also studied the size of the tumour, which was shown to be a relevant characteristic for recurrence ([HR]: 1.02; 95% CI: 8.3–9.6, *p* < 0.001) and specific‐cancer survival ([HR]: 1.0; 95% CI: 0.9–1.0, *p* < 0.001); however, there was no statistical correlation with Overall Survival, even when stratified by size thresholds (4, 7 and 10 cm).^18^

This study also has limitations. We presented a retrospective study with a single centre sample focused only on the Latin American population with a 6% dropout rate. However, this is a long‐term follow‐up series of patients with pT3aN0M0 RCC who underwent radical nephrectomy that we utilized to develop a helpful prognostic score to stratify postoperative risk, and that may have future utility within the context of new adjuvant therapies with anti‐programmed death 1 (PD‐1) for patients with pathological staging T3aN0MO.

## 5 CONCLUSIONS

Patients with the pT3aNo renal cell carcinoma is a non‐uniform group with different outcomes affected by preoperative weight loss, stage V chronic kidney disease, presence of sarcomatoid pattern and coagulative tumour necrosis. Our score offers a simplified risk‐stratification tool that accurately incorporates the relative contribution of both clinical and anatomopathological characteristics into overall survival prognosis after radical nephrectomy. This new instrument may prove helpful in routine clinical care, in stratifying patients to individualize surveillance and adjuvant therapy.

## AUTHOR CONTRIBUTIONS

**Caio Vinícius Suartz:** Conception of the idea, data collection, statistical analysis, handwriting. **Maurício Dener Cordeiro:** Conception of the idea and correction of the manuscript. **Paulo Afonso de Carvalho:** Correction of the manuscripts. **Fábio Pescarmona Gallucci:** Correction of the manuscripts. **Leopoldo Alves Ribeiro‐Filho:** Correction of the manuscripts. **Leonardo Cardili:** Correction of the manuscripts. **Arjun Sivaraman:** Correction of the manuscripts. **François Audenet:** Correction of the manuscripts. **José Maurício Mota:** Correction of the manuscripts. **William Carlos Nahas:** Correction of manuscripts and supervision.

## CONFLICT OF INTEREST STATEMENT

The authors declare no conflicts of interest.

## Supporting information


**Figure S1.** The receiver operating characteristic curve with area under the curve value of 0.7 (A) and Calibration plot to the score.

**Table S1.** ‐ Predictors of overall survival ‐ univariate analysis and multivariate analysis.
