# Supplementary material for: Scoring system for prediction of overall survival in patients with renal cell carcinoma T3aN0M0
Source: BJUI Compass. 2023 Nov 10;5(2):289–96. doi: 10.1002/bco2.309 (PMC10869657; doi:10.1002/bco2.309)

Supplementary figure 1. The receiver operating characteristic curve with area under the curve value of 0.7 (A) and Calibration plot to the score.

A

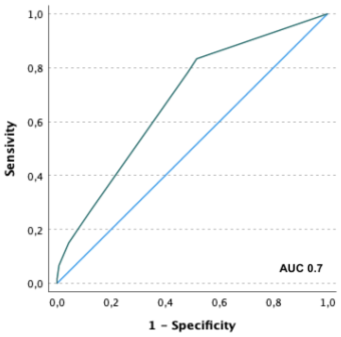

B

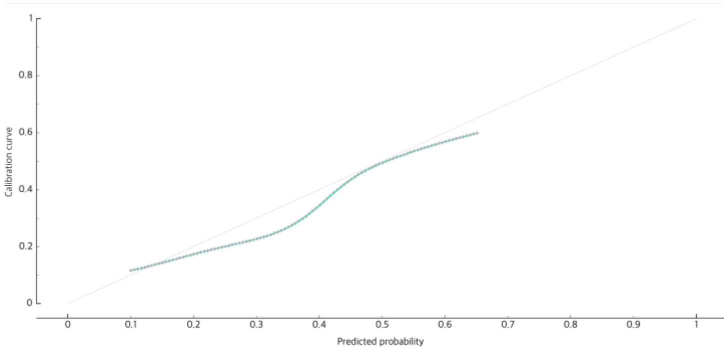

Supplement: Supplementary file 1 — Figure S1. The receiver operating characteristic curve with area under the curve value of 0.7 (A) and Calibration plot to the score. [file BCO2-5-289-s001.pdf]
