# Supplementary material for: Scoring system for prediction of overall survival in patients with renal cell carcinoma T3aN0M0
Source: BJUI Compass. 2023 Nov 10;5(2):289–96. doi: 10.1002/bco2.309 (PMC10869657; doi:10.1002/bco2.309)
Supplement: Supplementary file 2 — Table S1. ‐ Predictors of overall survival ‐ univariate analysis and multivariate analysis. [file BCO2-5-289-s002.docx]

|  |
| --- |

Supplementary table 1 - Predictors of overall survival - univariate analysis and multivariate analysis

|  |  |  |  |  |  |  |  |  |
| --- | --- | --- | --- | --- | --- | --- | --- | --- |
| **Univariate Analysis**  **Predictors of Overall Survival (OS)** | |  |  |  | **Multivariate Analysis**  **Predictors of Overall Survival (OS)** | | |  |
|  |  |  |  |  |  |  |  |  |
|  | **HR** | **C.I** | **P** |  |  | **HR** | **C.I** | **P** |
| **Tumoral size** | 1.0 | 0.9 - 1.0 | 0.144 |  |  |  |  |  |
| **Neutrophil lymphocyte ratio** | 0.2 | 0.9 - 1.1 | 0.286 |  |  |  |  |  |
| **BMI** | 0.9 | 0.8 - 1.0 | 0.055 |  |  |  |  |  |
| **Renal capsule invasion** | 1.0 | 0.5 - 1.7 | 0.020 |  | **Renal capsule invasion** | 1.3 | 0.7 - 2.5 | 0.376 |
| **Renal sinus fat invasion** | 1.1 | 0.5 - 1.8 | 0.002 |  | **Renal sinus fat invasion** | 1.1 | 0.5 - 2.4 | 0.671 |
| **Perirenal fat invasion** | 1.2 | 0.7 - 2.1 | 0.550 |  |  |  |  |  |
| **Renal sinus and perirrenal fat invasion** | 0.2 | 0.3 -1.1 | <0.001 |  | **Renal sinus and perirrenal fat invasion** | 0.4 | 0.1 - 1.2 | 0.278 |
| **Lymphovascular invasion** | 1.1 | 0.6 - 2.1 | 0.214 |  |  |  |  |  |
| **Clear cells histology** | 1.0 | 0.5 - 1.9 | 0.928 |  |  |  |  |  |
| **Sarcomatoid differentiation** | 5.5 | 2.3 - 13.1 | <0.001 |  | **Sarcomatoid differentiation** | 5.5 | 2.3 -13.1 | <0.001 |
| **Presence of coagulation necrosis** | 3.2 | 1.7 - 6.0 | <0.001 |  | **Presence of coagulation necrosis** | 3.2 | 1.7 - 6.0 | <0.001 |
| **Thrombus in renal vein** | 1.3 | 0.6 - 2.7 | 0.383 |  |  |  |  |  |
| **Weight loss** | 2.4 | 1.1 - 5.0 | 0.017 |  | **Weight loss** | 1.6 | 1.3 - 4.2 | 0.005 |
| **Genre** | 0.9 | 0.5 - 1.7 | 0.974 |  |  |  |  |  |
| **Fuhrman grade** | 1.9 | 1.2 - 2.9 | 0.020 |  | **Fuhrman grade** | 1.5 | 0.2 - 1.3 | 0.226 |
| **Age** | 0.8 | 0,9 - 1.1 | 0.686 |  |  |  |  |  |
| **Stage V chronic kidney disease** | 3.9 | 1.4 - 10.9 | 0.002 |  | **Stage V chronic kidney disease** | 2.2 | 1.2 - 5.0 | 0.017 |
|  |  |  |  |  |  |  |  |  |
